# The Staff Observation Aggression Scale – Revised for Ambulance Services (SOAS-RA)

**DOI:** 10.1186/s13049-025-01472-6

**Published:** 2025-10-09

**Authors:** May Elin Juliusdatter Haug, Elisabeth Haug, Roger Almvik, Tom Palmstierna, Hege Skundberg-Kletthagen

**Affiliations:** 1 Faculty of Medicine and Health, Institute of Health Sciences, Norwegian University of Science and Technology (NTNU), Gjøvik, Norway; 2 Division of Mental Health, Innlandet Hospital Trust, Brumunddal, Norway; 3 Department of Mental Health, Norwegian University of Science and Technology (NTNU), Trondheim, Norway; 4 Department of Clinical Neuroscience, Centre for Psychiatry Research, Karolinska Institute, Stockholm, Sweden

**Keywords:** Aggression, Ambulance services, Prehospital care, SOAS-R, SOAS-RA, Workplace violence

## Abstract

**Introduction:**

Ambulance personnel frequently encounter aggression in dynamic and unpredictable environments. Despite growing awareness of workplace violence in healthcare, few validated tools exist for systematic documentation in ambulance services.

**Objective:**

This study aimed to adapt and validate the Staff Observation Aggression Scale – Revised (SOAS-R) for use in ambulance services (SOAS-RA), and to examine the relationship between SOAS-RA severity scores and staff’s subjective perceptions of incident severity using a Visual Analogue Scale (VAS).

**Methods:**

Using a modified Delphi method, a panel of ambulance professionals adapted the SOAS-R to the ambulance service context. Data were collected from 34 ambulance stations across Norway using paper-based SOAS-RA forms. A total of 402 reports were submitted, with 302 including valid VAS scores. Descriptive and inferential statistical analyses examined associations between objective severity scores (SOAS-RA) and subjective ratings (VAS).

**Results:**

SOAS-RA total scores showed a small to moderate correlation with VAS ratings (*r* = 0.350, *p* < 0.001). The strongest predictor of perceived severity was “consequences for the victim” (β = 0.274, *p* < 0.001), followed by “means used by the aggressor” (β = 0.180, *p* < 0.001). Female staff rated incidents as more severe than male staff (*p* = 0.030), despite similar SOAS-RA scores.

**Conclusions:**

The SOAS-RA, combined with VAS, may serve as a valid, context-sensitive tool for documenting aggression in ambulance services. Future research should explore broader implementation and digital integration to enhance usability, data quality, and support organizational learning.

## Introduction

Workplace violence is a major and growing concern across numerous occupational sectors worldwide, with health services among the most affected [1, 2]. Within healthcare, staff working in mental health, emergency departments, and prehospital settings—such as ambulance services—are particularly vulnerable to violent incidents [3–7]. Ambulance staff frequently deliver care in unpredictable and uncontrolled environments, including private homes and public areas. These dynamic settings often involve limited patient information, time-critical decisions, and safety risks beyond the staff’s control [2, 8, 9].

Inspections and research have revealed considerable shortcomings in risk assessment, preventive planning, and the implementation of protective measures in ambulance services [10, 11]. Moreover, violence against ambulance staff is commonly underreported, which hinders systematic documentation and obstructs the development of effective safety measures [2, 3, 12–14].

Workplace violence is a complex and multifaceted phenomenon that encompasses verbal threats, physical assaults, and psychological or sexual harassment [8, 15]. It can result in significant physical, psychological, and emotional harm, and is linked to long-term consequences such as anxiety, stress, burnout, and absenteeism [16–20]. For healthcare organizations, this poses a dual challenge—protecting employee well-being while maintaining service continuity.

### The Norwegian ambulance service

The Norwegian ambulance service includes ground, boat, and air ambulances, and forms a vital component of the national emergency medical chain. Ambulance staff operate under various organizational models and handle a broad range of clinical presentations, from acute somatic illness and trauma to psychiatric crises and substance-related emergencies [10, 21]. These situations demand rapid clinical assessment and decision-making, often with little contextual information, and the expanding role of ambulance staff in community and primary care settings further illustrates the breadth and complexity of their responsibilities [22, 23]. In 2021, ambulance services carried out nearly 800,000 missions nationwide, 97% of which were conducted by ground ambulances [24]. Despite this high operational volume and clinical complexity, the sector still lacks context-appropriate and standardized tools for systematically recording and managing incidents of workplace violence.

### Research gap and rationale

Although workplace violence in healthcare has received increasing research attention, most studies have concentrated on institutional settings such as hospitals and psychiatric wards [5, 25]. In contrast, violence in ambulance services remains underexplored, and existing tools for documentation and risk assessment are poorly adapted to the decentralized and mobile nature of ambulance care [26–28].

The SOAS was originally developed by Palmstierna and Wistedt in the mid-1980s to document and assess the severity and characteristics of aggressive incidents in institutionalized psychiatric settings [29]. This tool was later refined into the SOAS-R, which introduced a more robust scoring structure to improve validity and usability [30]. Both instruments have undergone psychometric testing and demonstrated adequate reliability. The SOAS and its revised version, SOAS-R, were designed as structured, event-based reporting tools to document violent incidents shortly after they occur. This incident-based approach allows for systematic documentation while the details are still fresh in memory, improving the accuracy and completeness of incident data [29, 30]. Such a framework is particularly relevant in ambulance services, where dynamic and unpredictable environments require tools that can capture the complexity of violent encounters in real time, rather than relying on retrospective reporting systems.

The SOAS-R, in particular, has seen widespread use across various countries, primarily in Europe and parts of Asia, reflecting strong face validity [31, 32]. As of June 2025, the SOAS and SOAS-R collectively received 406 citations in the Web of Science, thereby underscoring their relevance in the field.

Several alternative instruments have been developed to assess aggression, including the Modified Overt Aggression Scale (MOAS) [33], which is widely used in psychiatric settings in the United States. Although MOAS demonstrates good psychometric properties, it is designed to measure cumulative aggression over a defined time frame rather than discrete incidents, which limits its utility in incident-based analysis. The Attempted and Actual Assault Scale (Attacks) [34] is another instrument designed for incident-level assessment, but its complexity has hindered widespread adoption. More recently, the Violent Event Severity Tool (VEST) [35] was introduced to categorize incidents based on type and severity. Although promising, it remains in the early stages of validation and lacks extensive psychometric evaluation.

The Aggressive Behaviour Risk Assessment Tool for Emergency Medical Services (ABRAT-EMS) is a brief screening tool designed to identify high-risk patients prior to contact [36]. Although ABRAT-EMS and VEST are helpful for risk prediction and incident classification, respectively, they do not offer a comprehensive framework for documenting and analysing the contextual nuances of violence encountered in dynamic prehospital environments.

Existing tools serve specific functions—such as aggregate scoring or risk screening—but fall short in capturing the complexity of real-time violent incidents in ambulance settings. This highlights a need for a dedicated instrument that integrates the validated structure of the SOAS-R with the operational demands of ambulance services.

Given its established psychometric strengths and structured, incident-based design, the SOAS-R provides a suitable foundation for adaptation to ambulance services. It has already demonstrated flexibility through successful use in emergency outpatient settings (SOAS-RE) [37], further supporting its relevance for dynamic, time-sensitive contexts such as ambulance services. The Staff Observation Aggression Scale – Revised for Ambulance Services (SOAS-RA) is based on the original SOAS developed by Palmstierna and Wistedt [29] and the revised SOAS-R by Nijman et al. (1999). These tools are not intended as predictive risk-assessment instruments but rather as standardized frameworks for recording the characteristics and severity of violent incidents when they occur. This approach enables systematic reporting of aggressive events shortly after they happen, thereby ensuring that details are captured accurately and reliably while the event is fresh in the memory. Recent adaptations such as the SOAS-RE for emergency primary health care [21] follow the same principle of structured real-time documentation rather than retrospective reviews or risk stratification. Similarly, the SOAS-RA has been developed to fit the context of ambulance services, providing a reliable tool for timely documentation of workplace violence experienced by ambulance personnel.

The intended use of SOAS-RA is as a structured, post-incident documentation tool, designed to capture the characteristics and severity of violent events experienced by ambulance staff. It is not designed for risk prevention or prevention prior to incidents but rather to support accurate reporting, organizational learning, and staff support following violent encounters.

To address the identified gaps, the present study aimed to adapt and validate the SOAS-R for use in ambulance services. Additionally, the study examined how severity scores from the SOAS-RA aligned with ambulance personnel’s subjective perceptions of incident severity, as measured by a Visual Analogue Scale (VAS). The original SOAS-R scoring system was retained to ensure consistency in severity assessment.

## Method

### Design and adaptation process

This study employed a structured, stepwise adaptation of the Staff Observation Aggression Scale – Revised (SOAS-R) for use in ambulance services. The resulting tool, the Staff Observation Aggression Scale – Revised for Ambulance Services (SOAS-RA), was developed using a modified Delphi technique. This method is particularly suitable in contexts where empirical evidence is limited and expert consensus is required to ensure contextual and content validity [38, 39].

The adaptation process was informed by comparable applications of the Delphi method in ambulance settings, such as those by Richards et al. [40] and the competency framework for paramedics developed by Tanninen et al. [41]. These studies demonstrate the value of Delphi-based consensus-building in the creation of practical tools aligned with the operational realities of emergency medical services.

### Expert panel and development process

An expert panel comprising 11 professionals from Norwegian ambulance and healthcare services was recruited to ensure both clinical and academic breadth. The panel included nine certified ambulance practitioners, several of whom held additional qualifications as registered nurses (*n* = 7), anaesthesia nurses (*n* = 2), and master’s degrees in clinical nursing or prehospital care (*n* = 3). The participants represented a range of professional roles, including operational managers, training coordinators, emergency driving instructors, department heads, and academic positions (including one doctoral research fellow and one university lecturer leading a simulation centre). All participants had a minimum of five years’ experience within ambulance or acute healthcare services, and were selected based on a combination of criteria encompassing practical and academic expertise and educational background. Participation was voluntary and based on informed consent, which was considered granted through the participants’ active feedback and contributions to the development of the questionnaire**.**

### Overview of study phases

The adaptation and validation of the SOAS-RA followed a structured, three-phase process. First, a modified Delphi method was used to revise and tailor the original SOAS-R items to the ambulance service context. Second, the preliminary version was pilot-tested at 12 ambulance bases to assess face validity and practical applicability. Third, the revised tool was rolled out across 34 ambulance stations for full-scale data collection, including use of the SOAS-RA in combination with a Visual Analogue Scale (VAS) to assess construct and concurrent validity. The following sections describe each of these phases in greater detail.

### Delphi procedure

This study employed a three-phase modified Delphi process to adapt the SOAS-R instrument to ambulance services. The procedure consisted of:

1. an initial exploratory phase with qualitative input and suggested revisions (Round 1),
2. a revision and re-distribution phase with more structured content (Round 2), and
3. a final consensus phase involving face and content validation in a physical meeting (Round 3).

Each phase built iteratively on the previous one, following key principles of the Delphi method such as controlled feedback, anonymity, and repeated review rounds. Below, each round is described in more detail.

Round 1: The panel reviewed the original SOAS-R and proposed adaptations to reflect the ambulance context. Terminology, item structure, and contextual relevance were revised. The adaptation followed a modified Delphi process characterized by iterative revision, anonymity, and controlled feedback, consistent with qualitative Delphi approaches [38, 42]. Time of incident was recoded into broader categories (e.g., shift, weekday, month), based on feedback from the Data Protection Official (SIKT).

Round 2: A revised draft of the SOAS-RA was distributed. This version included expanded categories for incident location (e.g., public place, private residence, emergency department), as well as background variables concerning the patient’s mental state, intoxication, and role of the aggressor. Core SOAS-R domains (provocation, type of aggression, target, consequences, intervention) were adjusted to fit the ambulance setting.

Round 3: The final version was assessed in a physical meeting to evaluate face and content validity. As no further suggestions emerged, consensus was deemed to have been achieved.

All feedback was analysed and summarized by the research team and redistributed to the panel members, in line with the principles of the Delphi methodology [38].

### Reference to original instrument

The SOAS-RA was adapted from the validated original version of the SOAS-R [30].

### Pilot-testing

The SOAS-RA was piloted at 12 ambulance stations that had already been recruited to the project and therefore served as the test group for the initial practical trial of the instrument. A total of 47 forms were completed following incidents involving aggression. To assess the clarity, usability, and content relevance of the form, structured debriefings were conducted with staff at each station. Based on the feedback received, no further revisions were deemed necessary, in accordance with recommendations regarding pilot-testing as part of quality assurance prior to broader implementation [39].

### Scoring system

The SOAS-RA retains the original SOAS-R scoring methodology [21, 30], where severity is determined by summing the maximum points from five core categories. The SOAS-R total severity score ranges from 0 to 22 points. A total score of 16 or more indicates a severe incident. In addition, a Visual Analogue Scale (VAS) (0–100 mm) was used to capture the responder’s subjective perception of severity.

### Participants

The study involved ambulance personnel from 34 of the 299 ambulance stations across Norway. All participating stations were invited to contribute based on their operational relevance and distribution across various regions. All on-duty employees at the selected stations were encouraged to complete the SOAS-RA form following incidents involving aggression or violence. Participation was entirely voluntary and anonymous. The sample included a broad representation of ambulance staff, thereby supporting the contextual validity of the instrument within real-world ambulance service settings.

### Study sites

Study sites were selected in collaboration with regional health authorities to ensure variation across geographical regions, urban and rural contexts, and organizational structures. Although the sampling was purposive rather than stratified, this approach aimed to enhance the ecological and operational representativeness of the findings.

### Recruitment

Recruitment was conducted in collaboration with regional managers across various health trusts to ensure geographical and organizational diversity. Information about the study was disseminated through national conferences, regional seminars, and existing professional networks. The first author visited each selected station to deliver in-person information sessions and provide training. These sessions were complemented by digital meetings and a standardized instructional video, which was distributed to local contact persons. Continued communication was maintained throughout the 12-month data collection period to support engagement, protocol adherence, and high data quality.

### Data collection

Data were collected over a 12-month period. Participants completed the SOAS-RA form immediately after experiencing incidents involving aggression or violence, typically upon returning to the ambulance station at the end of their shift.

A total of 402 SOAS-RA forms were submitted, representing 310 unique incidents. In 92 cases, more than one staff member independently completed a form for the same event. This resulted in multiple subjective assessments of the same incident, which were treated as separate observations in the analyses.

### Statistical analyses

Background variables were summarized using means, medians, standard deviations, and percentage distributions. SOAS-RA total scores and VAS scores were analysed using means, medians, and interquartile ranges (IQR) to capture distribution characteristics. Pearson’s correlation coefficient was used to examine the relationship between SOAS-RA and VAS scores. Multiple linear regression analysis was performed to assess the contribution of each SOAS-RA domain to the VAS severity score. To assess the assumptions of linear regression, multicollinearity was evaluated using Variance Inflation Factor (VIF), with all values below 1.1. Residual independence was assessed using the Durbin–Watson statistic, which suggested potential dependency between observations. ANOVA was used to explore differences in severity ratings by age group and gender. All statistical analyses were conducted using IBM SPSS Statistics version 30.0.0.0 (IBM Corp., Armonk, NY), with a two-tailed significance level set at *p* ≤ 0.05.

## Results

### Participation and descriptive statistics

A total of 34 ambulance stations participated in the study, representing both urban and rural areas. Station size varied from one to eight vehicles in 24-h service. In total, 402 completed SOAS-RA forms were submitted and included in the descriptive analysis. Of these, 302 forms contained complete Visual Analogue Scale (VAS) data and were used in analyses. An overview of ambulance staff characteristics, incident details, and aggressor profiles is presented in Table 1.

**Table 1:** Background characteristics of ambulance staff, incident details, and aggressor characteristics encountered

Characteristic	Value
Staff Background	
Age (*n* = 391)	Median: 31 (Min–Max: 18–67)
Number of years in the service (*n* = 396)	Median: 6 (Min–Max: 0–44)
Gender	
Male	203 (50.5%)
Female	197 (49.0%)
Educational Background	
Ambulance Technician or Emergency Medical Technician	234 (58.2%)
Other healthcare professions (e.g., paramedic, nurse)	162 (40.3%)
Incident Details	
Time	
Monday-Thursday	251 (62.4%)
Friday-Sunday	151 (37.6%)
Shift	
Day	149 (37.1%)
Evening	120 (29.9%)
Night	129 (32.1%)
Location of Incident	
Emergency department	56 (13.9%)
Home address	131 (32.6%)
In public spaces	157 (39.1%)
Other (e.g., in the ambulance)	57 (14.2%)
Alone During the Incident	
Yes	14 (3.5%)
No	388 (96.5%)
Aggressor Characteristics	
Gender	
Male	254 (63.2%)
Female	139 (34.6%)
Role of the Aggressor	
Patient	378 (94%)
Next of kin	9 (2.2%)
Known Mental Disorder	
Yes	134 (33.4%)
No	268 (66.6%)
Substance Abuse	
Yes	244 (60.7%)
No	94 (23.4%)
Unknown	64 (15.9%)
Opportunity to Receive More Information About the Patient	
Yes	72 (17.9%)
No	320 (76.9%)

Site, time and characteristics of the persons involved in the aggressive incidents (n = 402). Due to missing data on some items, the numbers do not add up to the total of 402

Of the total of *n* = 162 participants in the “other healthcare professions” group, *n* = 111 were ambulance technicians with additional qualifications through the national paramedic education and/or specialized training in addiction and psychiatry. A further *n* = 18 had completed a bachelor’s degree in paramedicine, *n* = 22 were registered nurses, *n* = 9 were students or apprentices in paramedicine, ambulance care, nursing, or medicine, and *n* = 2 were observers.

**Fig. 1:**
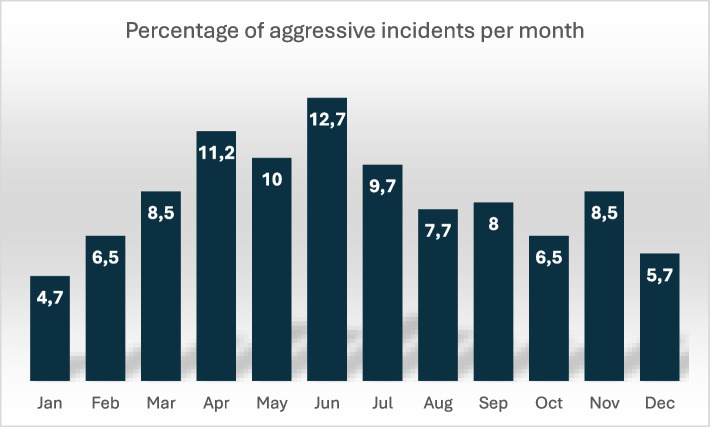
Monthly distribution of reported aggressive incidents in ambulance services (%)

Of the incidents classified as occurring in “other places” (*n* = 57), the majority took place inside or immediately outside the ambulance (*n* = 42). Additional incidents occurred at addiction and psychiatric institutions (*n* = 9), nursing homes (*n* = 4), and police custody facilities (*n* = 2). The incidents were distributed as follows throughout the year (see Fig. 1).

**Fig. 2:**
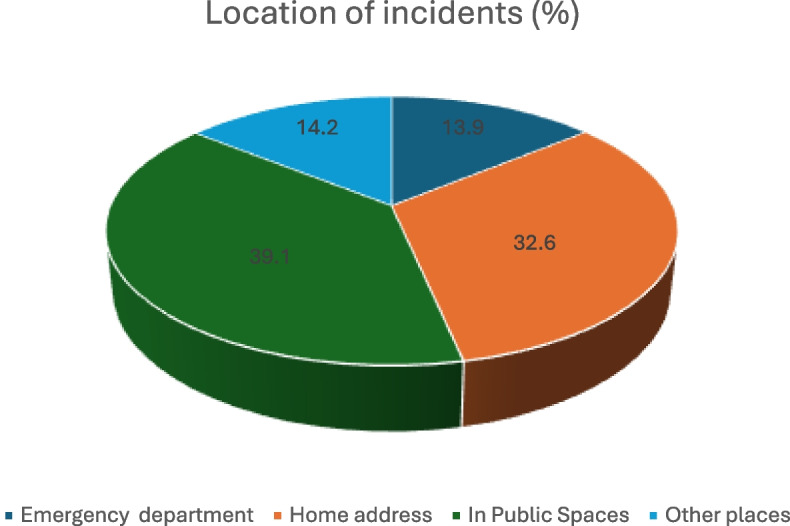
Distribution of incident locations across different settings

The incidents occurred across different settings, as illustrated in Fig. 2. Of the incidents classified as occurring in ‘other places’ (*n* = 57), the majority took place inside or immediately outside the ambulance (n = 42). Additional incidents occurred at addiction and psychiatric institutions (*n* = 9), nursing homes (*n* = 4), and police custody facilities (*n* = 2).

### SOAS-RA and VAS scoring results

**Fig. 3:**
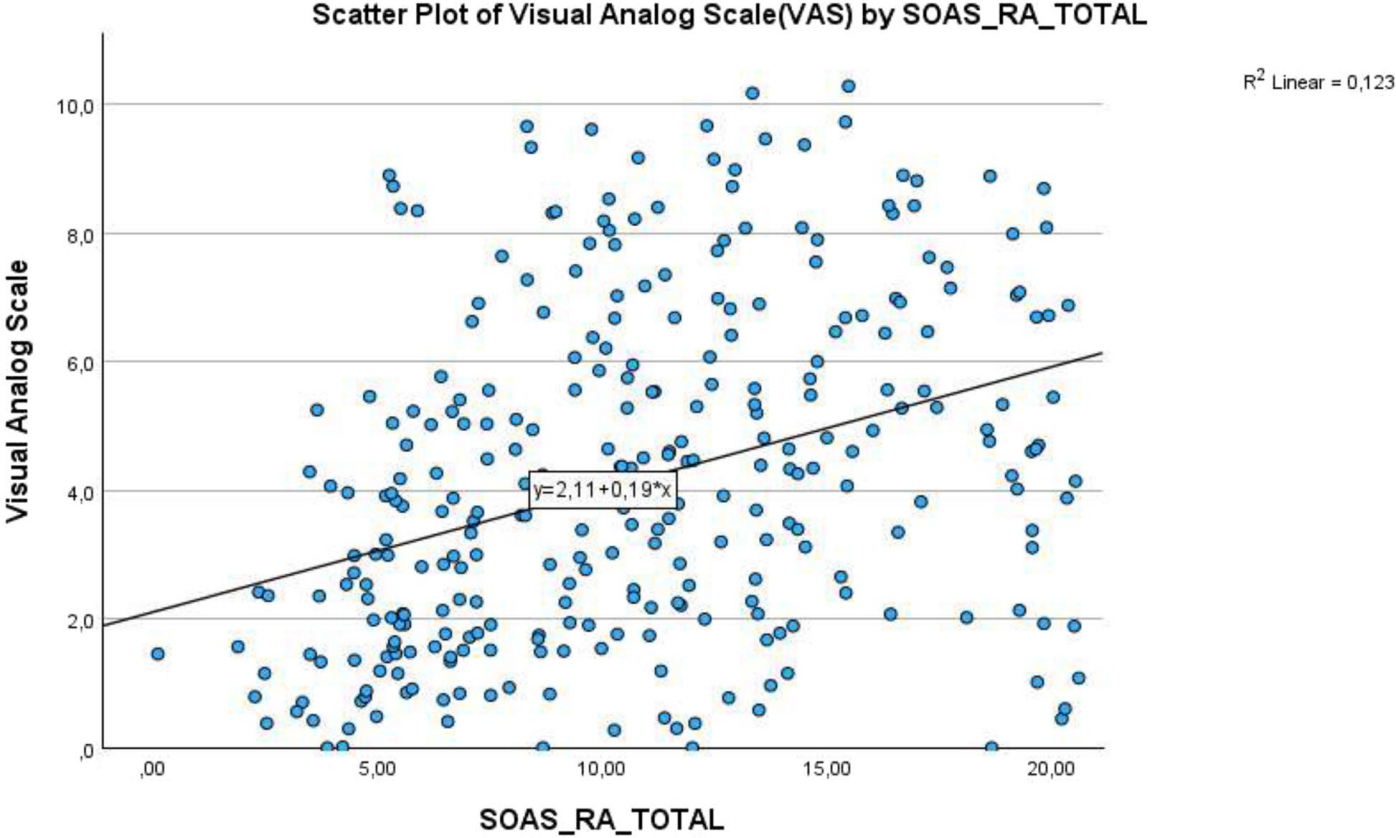
Scatterplot of Visual Analogue Scale (VAS) by SOAS-RA Total

Scatterplot showing the relationship between SOAS-RA total severity scores and Visual Analogue Scale (VAS) severity scores, with a linear trendline indicating a modest positive association (Fig. 3).

The total severity score on the SOAS-RA ranged from 0 to 20, with a mean of 10.78 (SD = 4.78). The distribution was approximately symmetrical (skewness = 0.29) but revealed both floor and ceiling effects (i.e., scores of 0 and 20).

VAS severity scores ranged from 0 to 10, with a mean of 4.17 (SD = 2.61), and slight positive skewness (0.35). A pronounced floor effect was observed, with several cases receiving a score of 0.

### Correlation between SOAS-RA and VAS

A small to moderate positive correlation was found between the SOAS-RA total score and the VAS rating (*r* = 0.350, *p* < 0.001), indicating a statistically significant association between objectively rated severity and subjective perception of seriousness.

No statistically significant differences were found in SOAS-RA or VAS scores based on age (*p* = 0.094 and *p* = 0.254, respectively). However, a significant gender difference was observed for VAS scores (*p* = 0.030), which suggests that female participants rated incidents as more severe than their male counterparts, despite no difference in objective severity scores.

Table 2 presents the SOAS-RA severity points, the mean SOAS total score with corresponding standard deviations, and the mean VAS score (*n* = 302).

**Table 2:** SOAS-RA severity points, mean SOAS total score, and standard deviations, mean VAS scores, standard deviations (*n* = 302)

		SOAS-RA Severity Total Score^a^	SOAS-RA Points Assigned^b^	VAS Severity Score
SOAS-RA columns:		Mean (SD)		Mean (SD)
1. Provocation	n			
No understandable provocation	112	11.1 (4.5)	1	46.9 (26.9)
Had to wait	23	9.9 (4.5)	0	42.5 (25.4)
Was denied something	41	11.2 (5.0)	0	47.6 (26.6)
Disagreed with the assessment	93	11.45 (5.1)	0	39.4 (22.8)
Involuntary assessment of health status	67	11.8 (5.1)	2	39.1 (22.2)
Opposed transport	79	11.9 (5.0)	2	39.7 (21.6)
2. Means Used by Aggressor				
Verbal aggression	195	11.2 (4.8)	0	41.2 (24.7)
Threat of violence	118	11.3 (4.8)	1	45.8 (26.7)
Threat of harm	129	11.7 (4.7)	1	47.2 (26.7)
Threat of killing	55	11.4 (5.2)	1	47.6 (28.3)
Threat of a sexual nature	16	12.3 (3.7)	1	39.5 (25.0)
Body-part hand	165	11.8 (4.8)	2	42.2 (25.7)
Body-part foot	77	13.2 (4.7)	2	52.1 (24.4)
Body-part head	27	14.9 (4.2)	2	53.3 (9.2)
Body-part spitting	47	13.3 (4.6)	2	51.8 (24.9)
Objects/furniture/equipment	59	11.8 (4.9)	1	47.4 (28.3)
Attempted strangulation	4	16.5 (4.0)	3	52.5 (46.3)
Used/had a weapon	19	13.1 (4.9)	3	54.3 (33.4)
Other dangerous objects, including syringe	12	12.1 (4.4)	3	47.1 (22.1)
3. Target of Aggression/Victim				
Nothing/nobody	12	6.6 (4.9)	0	40.9 (27.9)
The patient themselves/self-harm	47	12.5 (5.0)	3	47.2 (28.3)
Items/furniture/equipment	81	12.8 (4.6)	1	48.7 (25.1)
Doctor	41	11.1 (4.6)	3	44.5(22.9)
Ambulance crew	236	11.1 (4.6)	3	41.7 (24.7)
Police	84	12.9 (4.8)	3	53.4 (24.5)
4. Consequence(s) for Victim(s)				
None	134	7.4 (3.4)	0	30.0 (21.3)
Object(s) damaged	32	13.4 (3.9)	3	58.3(24.8)
Object(s) destroyed	24	13.9 (3.3)	3	63.9 (18.5)
Psychological/emotional stress	87	13.6 (3.8)	4	53.9 (24.9)
Felt their own safety threatened	103	13.3 (3.2)	6	54.2 (25.2)
Physical pain	43	17.1 (2.8)	9	54.8 (24.9)
Visible injury	22	18.1 (2.2)	9	56.7 (25.4)
Need for medical treatment/first aid	12	16.7 (2.5)	9	59.6 (21.4)
Need to be taken off duty	8	17.1 (2.9)	9	33.4 (29.5)
5. Measure(s) to Stop Aggression				
None	10	8.4 (2.7)	0	15.0 (14.6)
Talked to the person	223	10.5 (4.7)	0	40.0 (24.7)
Took the person aside	22	11.1 (4.6)	0	45.9 (21.9)
Withdrew from the situation	120	10.7 (4.1)	0	45.7 (24.8)
Complied with the person’s request	19	9.6 (4.1)	0	47.7 (26.1)
Restraint	81	15.3 (4.3)	4	47.7 (24.9)
Medication	14	12.7 (4.8)	2	72.5 (16.7)
Took the person to the vehicle by force	43	14.8 (4.2)	4	51.1 (22.2)
Allowed the patient to leave the ambulance	13	10.3 (4.9)	4	54.1 (30.2)

^a^Mean SOAS-RA total score: This represents the average total score for all incidents in which this category was reported. Even if a category has a low assigned score, the mean total score may be high because these incidents often involve other severe factors (e.g., physical violence or significant consequences for the victim)

^b^Assigned SOAS-RA points: These are the predefined scores assigned to each variable in the SOAS-RA scale. They represent the fixed weighting of the severity of an incident within each category

### Contribution of SOAS-RA dimensions to VAS scores

A multiple linear regression analysis examined the contribution of individual SOAS-RA dimensions to VAS scores (*n* = 302). The results are summarized in Table 3.

**Table 3:** Standardized Beta coefficients and explained variance for VAS score estimated from SOAS-RA column score by a multiple linear regression (*n* = 302)

SOAS-RA Column	*Standardized Beta*	*Explained Variance*	*P-Value*
1. Provocation	0.032	−0.003	0.567
2. Means used by aggressor	0.180	0.053	< 0.001
*3. Target of aggression/victim*	0.050	0.055	0.360
4. Consequences for victim(s)	0.274	0.130	< 0.001
5. Measures to stop aggression	0.054	0.130	0.340

A multiple linear regression analysis revealed that the overall model was statistically significant and explained a modest but meaningful portion of the variance in perceived severity scores (VAS). Among the five SOAS-RA dimensions, two emerged as statistically significant predictors: the means used by the aggressor and the consequences for the victim. The remaining variables showed no significant relationship with perceived severity. The findings were supported by the analysis using robust standard errors.

### Descriptive results by SOAS-RA categories

Detailed descriptive statistics (means and SDs) for SOAS-RA and VAS scores across subcategories (e.g., types of provocation, forms of aggression, intervention methods) are presented in Table 2.

The highest VAS scores were associated with physical harm, visible injury, need for medical treatment, and use of weapons or dangerous objects.

Incidents involving spitting, attempted strangulation, or headbutting also scored highly.

Low-severity VAS ratings were typically linked to verbal aggression, waiting-related provocations, and non-confrontational resolution strategies (e.g., talking or withdrawing).

There was no consistent relationship between the method used to de-escalate and the perceived severity of the incident, which suggests that severity perception is shaped more by impact than by intervention.

## Discussion

The primary aim of this study was to adapt and validate the Staff Observation Aggression Scale – Revised (SOAS-R) for use in ambulance services. This led to the development of the Staff Observation Aggression Scale – Revised Ambulance (SOAS-RA), which is a context-sensitive tool for documenting and assessing aggression and violence against ambulance staff. The study also examined the relationship between objective severity scores (SOAS-RA) and subjective perceptions of severity (VAS).

The findings indicate a small to moderate positive association between SOAS-RA total scores and VAS ratings, consistent with previous validation efforts in emergency primary healthcare and psychiatric contexts [21, 30]. This level of correlation is to be expected when comparing structured, incident-based scoring with subjective severity ratings.

### Consequences for the victim

The strongest predictor of high VAS scores was “consequences for victim(s)”, which indicates that the subjective perception of severity is most influenced by the immediate personal impact of the incident—whether physical, psychological, or emotional. It is also in line with the findings corroborating the SOAS-R development where “consequences for victim(s)” was also the strongest correlation to perceived severity [30]. This aligns with prior research emphasizing the link between exposure to aggression and outcomes such as anxiety, fear, and psychological distress [16, 30, 43].

These findings underscore the need for a victim-centred approach to both incident reporting and follow-up strategies, with a focus on individual experience and post-incident support.

Whilst the SOAS-RA provides a structured account of incident characteristics, the VAS captures personal emotional responses. Together, they offer complementary perspectives on severity.

### Aggressor’s methods and perceived severity

The methods used by the aggressor were also significantly associated with VAS ratings, although to a lesser extent. Violent actions such as spitting, use of weapons, or physical force were perceived as more severe, which confirms prior findings that the nature and intensity of the aggression shapes the psychological impact on victims [21, 44].

### Provocation and target of aggression

Neither provocation nor the target of aggression showed a statistically significant correlation with VAS scores. This suggests that the way in which an incident unfolds—rather than who or what is targeted or what initially triggered the aggression—has greater influence on how serious the event is perceived to be. These findings align with earlier studies indicating that the consequences of an incident, more than contextual cues, tend to shape perceptions of harm [45]. Moreover, the observed lack of measurable effect may reflect methodological limitations, such as the absence of adjustments for moderating factors such as previous exposure to violence or perceived organizational support. Research by DeLong and Reichert [46] supports the notion that individual background factors and accumulated experiences can significantly influence how people perceive, react to, or participate in aggressive encounters. Further studies are therefore needed to explore these potential indirect effects, particularly in complex settings such as ambulance emergency care, where both subjective and contextual factors interact dynamically.

### Intervention measures and VAS

No significant association was found between the intervention measures used and perceived severity. One explanation may be that most incidents were managed with low-intensity approaches (e.g., verbal de-escalation), whilst more coercive measures (e.g., medication, restraint) were rarely used and may have been applied as routine rather than reactive responses.

This is consistent with studies highlighting that de-escalation techniques are often implemented pre-emptively, and that their effectiveness may not always correspond directly to the perceived severity of the incident [47–49].

### Strengths of the study

One methodological strength of this study is the use of the SOAS-RA as an event-based documentation tool. The SOAS-RA offers several advantages over retrospective reporting systems that are commonly used for incident documentation in healthcare. By enabling structured, incident-based reporting shortly after the event occurs, the tool reduces recall bias and enhances data quality. Unlike general adverse event systems, which often capture only the most severe incidents, the SOAS-RA systematically records the full spectrum of violent encounters, including verbal threats and minor physical aggression. This provides a more comprehensive understanding of workplace violence, which is essential for targeted prevention strategies and operational planning in ambulance services.

This study further contributes methodologically by adapting an established assessment tool to a field where violence is both common and underreported, and where validated tools have been lacking. The adaptation process, guided by a modified Delphi technique, included a geographically and professionally diverse panel of ambulance staff, thereby enhancing the contextual relevance of the instrument.

Additionally, both SOAS-RA and VAS are user-friendly, low-resource tools that can be implemented in time-sensitive ambulance environments without compromising data quality. Their combination—capturing both objective incident characteristics and subjective severity assessments—offers a robust foundation for systematic incident analysis, organizational learning, and informed decision-making. The SOAS-RA can support ambulance services in identifying patterns of workplace violence, tailoring support to individual needs, and guiding prevention strategies.

A possible strength of the study is the inclusion of multiple reports from different staff members on the same incident. This may offer a unique opportunity to explore variation in perceived severity, and to illustrate the subjective and individual nature of threat perception.

From a practical perspective, this supports the need for post-incident procedures that are responsive to individual experiences, rather than assuming a shared perception within a team. The observed gender difference in perceived severity may reflect underlying gender-based differences in threat perception and emotional response. This pattern aligns with previous research on workplace violence in prehospital and emergency care settings. Female healthcare workers are disproportionately exposed to various forms of workplace violence, including verbal abuse and sexual harassment, which often result in significant psychological distress[50]. Similarly, female ambulance staff report higher levels of emotional strain and more frequent experiences of sexual harassment compared to their male colleagues[51]. These findings underscore the importance of incorporating gender-sensitive perspectives into safety protocols, post-incident support systems, and resilience-building strategies within emergency medical services.

### Limitations

Although the study followed a structured approach, several methodological considerations must be acknowledged. A key limitation concerns the potential dependency between observations. Of the 402 submitted SOAS-RA forms, these represented 310 unique incidents. In 92 cases, the same incident was independently reported by more than one staff member, in accordance with the instruction to document each person’s own experience. Additionally, some individuals submitted multiple forms throughout the 12-month data collection period. This may have introduced clustering both within incidents and within individual respondents. As the dataset did not include personal identifiers, hierarchical models or repeated-measures adjustments could not be applied [52]. Although this may have reduced the precision of regression estimates, the aim was to explore individual perceptions of severity. Therefore, each form was treated as an independent and meaningful observation.

Interrater reliability was not formally tested, which limits the ability to evaluate the consistency of scoring across users. Although written instructions and training were provided, future research should include formal reliability testing to strengthen the robustness of the instrument and ensure comparability across users and settings [53].

The sample was recruited through purposive collaboration with regional health authorities. Whilst this ensured operational diversity, it may limit the generalizability of findings. Voluntary participation also introduces a potential self-selection bias. Another limitation is the potential for reporting bias, as participants may have been more inclined to document incidents that they subjectively perceived as particularly severe or distressing. This selective reporting could have contributed to an overrepresentation of more serious cases, and may have artificially inflated the observed correlation between SOAS-RA and VAS scores.

Data were collected using paper-based forms, which may have contributed to underreporting or inconsistent documentation. Structural barriers such as the absence of systematic feedback and support systems are known to negatively affect reporting compliance [54]. Emerging research highlights that digital reporting platforms, especially when combined with training and feedback, may improve both reporting frequency and data quality [55]. Future studies should consider digital solutions with automated reminders (e.g., SMS or email) to support user engagement and data reliability in future applications of the SOAS-RA in ambulance services.

### Implications for practice

The SOAS-RA and VAS can support the systematic documentation, monitoring, and follow-up of violent incidents in ambulance services. The tools have the potential to increase awareness, improve reporting culture, and guide targeted interventions for affected personnel.

Implementation should be supported by integration into existing workplace health and safety systems, training, and a clear strategy for follow-up and feedback.

## Conclusion

The SOAS-RA, when used alongside the VAS, demonstrates potential as a valid, context-sensitive tool for documenting and assessing incidents of aggression in ambulance services. The findings indicate that perceived severity is most strongly influenced by the consequences experienced by staff, such as physical or psychological harm, rather than by provocation or professional role.

Whilst this relationship may seem intuitive, the study contributes conceptually by identifying which event characteristics are associated with stronger subjective threat responses. This perspective helps to clarify *how* staff interpret and respond to different forms of aggression.

Rather than aiming to predict which missions carry the greatest risk of violence, the study highlights which types of incidents are *experienced* as most harmful. These insights may inform more tailored post-incident support, strengthen reflective practice, and support organizational learning.

Thus, the integration of tools such as SOAS-RA and VAS can support ambulance services in understanding staff experiences of violence, improving reporting culture, and guiding both preventive strategies and individualized follow-up.

## Data Availability

No datasets were generated or analysed during the current study.
